# Evidence against a geographic gradient of Alzheimer's disease and the hygiene hypothesis

**DOI:** 10.1093/emph/eoaa023

**Published:** 2020-07-10

**Authors:** J Constance Lathe, Richard Lathe

**Affiliations:** e1 Program in Neuroscience, University of Glasgow, Glasgow, UK; e2 Division of Infection Medicine, University of Edinburgh Medical School, Edinburgh, UK

**Keywords:** Alzheimer, dementia, global burden of disease, hygiene hypothesis, prevalence

## Abstract

A significant positive correlation was previously reported (Fox *et al.Evol Med Public Health* 2013; **2013**:173–86) between hygiene and the global prevalence of Alzheimer's disease (AD) based on World Health Organization (2004) data. These data have now been updated by the Global Burden of Disease (GBD; 2016) dataset that takes into account under-registration and other potential confounds. We therefore addressed whether the association between hygiene and AD is maintained in light of these more recent data. We report a significant positive correlation between GBD AD prevalence rates and parasite burden, and a negative association with hygiene. These newer data argue that hygiene is not a risk factor for AD, and instead suggest that parasite burden may increase AD risk.

Lay summary: It was previously hypothesized that hygeine might be a risk factor for the development of Alzheimer disease (AD), based on a global gradient of dementia. Newer data that correct global AD rates for under-reporting now demonstrate that parasite burden is positively correlated with AD.

## GLOBAL GRADIENT OF ALZHEIMER'S DISEASE AND THE HYGIENE HYPOTHESIS

Fox *et al.* [1] reported a significant positive correlation between hygiene and Alzheimer's disease (AD) prevalence across the world. Their ‘hygiene hypothesis’ was rooted on geographic gradients in the worldwide rates of AD, in which developed countries with both strong hygiene and high rates of AD are at one end of the spectrum, whereas developing countries with poor hygiene and lower rates of AD are at the other.

The immune system (and by implication hygiene) undoubtedly plays a key role in AD. Patient brain is reported to contain elevated levels of microbes, and the biomarker of the disease, Aβ, is likely to be a defense protein induced by infection, underlining the contention that microbial infection/reactivation might be causally linked to AD development [2, 3]. In this case, prior exposure to a diversity of environmental microbes might potentially protect against AD, whereas hygiene might increase the risk.

The hygiene hypothesis has been invoked to explain differential rates of autoimmune disease across the world [4, 5]. Nevertheless, the underlying mechanisms are not well defined. There are several possibilities. First, hygiene (restricted exposure to a broad range of environmental microbes) could lead to failure of immunological tolerance, promoting inflammatory disease upon exposure to otherwise innocuous (or self) antigens. This is thought to underlie the association between living on traditional farms and protection from allergic conditions such as asthma (e.g. Ref. [6]). Conversely, in the absence of microbe exposure, responses to cross-reacting species are absent, and individuals may become susceptible to environmental pathogens. For example, infection with poxvirus protects against a second unrelated pathogen, herpes simplex [7], and mice rendered bacteria-free by streptomycin become highly susceptible to *Salmonella enterica* [8]; further examples are reviewed by Roossinck [9]. By either mechanism, restricted exposure to a spectrum of environmental microbes could predispose to some types of infectious/inflammatory disease.

The hypothesis that hygiene might be a risk factor for AD development is therefore plausible. However, Fox *et al.* base their conclusions on correlations between measures of infection such as parasite stress versus per-country rates of AD across the world, as reported by the World Health Organization (WHO) in 2004. Nonetheless, this dataset has been replaced by the Global Burden of Disease (GBD) dataset. GBD is a global initiative led by the Institute of Health Metrix and Evaluation (IHME) and is now under the aegis of both the IHME and the WHO [10]. GBD records differ from WHO datasets in that they involve systematic collation of all available data, corrections for under-registration, and model-fitting to remove inconsistencies (supplementary appendix to Ref. [11]). To address whether the hygiene hypothesis is consistent with these more recent data, we studied whether the correlation observed by Fox *et al.* is reproduced with the GBD dataset.

## METHODS: RE-EVALUATING THE HYGIENE HYPOTHESIS

The two most authoritative age-adjusted datasets are the WHO 2004 dataset [12] (https://www.who.int/healthinfo/global_burden_disease/estimates/en/index1.html, also https://www.who.int/healthinfo/global_burden_disease/GBD_report_2004update_full.pdf), that we refer to here as WHO2004, and the Global Burden of Disease (GBD) 2016 dataset [11] revised 2017 (available online at http://ghdx.healthdata.org/gbd-results-tool), that we refer to here as GBD2016.

As argued by Fox *et al.* [1], age-adjusted DALYs (disability-adjusted life years) per 100 000 population, that we define here as ADadj, are probably the best measure of the overall burden of AD in each country, and these were analyzed in their paper. The GBD output is also in age-adjusted DALYs per 100 000 population and is thus comparable to the WHO data. Moreover, although statistics generally report rates of untyped age-related ‘dementia’, AD is the predominant type of dementia, and both WHO and GBD (see below) address this slightly broader category (AD and other dementias); their results are therefore strictly comparable.

To address potential differences between the datasets we first compared the GBD ADadj values to the WHO dataset. We then reanalyzed the per-country parasite stress values (as given in figure 1 of Fox *et al.*) versus rates of AD (ADadj) in the GBD2016 dataset. Pearson's correlation was used to assess the statistical relationship between the two parameters.

**Figure 1.**
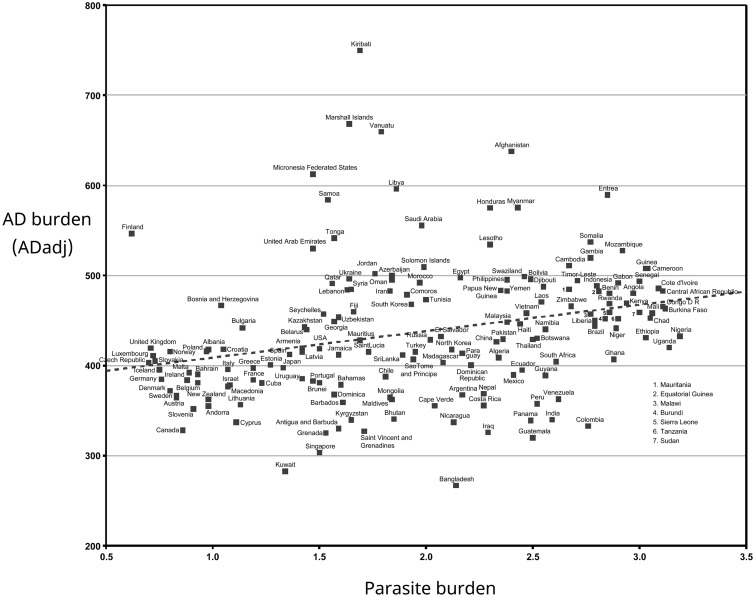
Parasite stress correlates positively with burden of Alzheimer's disease (AD). Plot of Global Burden of Disease (GBD2016, revised 2017) age-normalized DALYs (disability-adjusted life years) for AD per 100 000 Population (ADadj), against square root transformed values of contemporary parasite stress (transformed values and units according to Ref. [1]). Trendline added (Microsoft Excel). ADadj and parasite burden are positively correlated [*r*(166) = 0.28, *P *=* *0.0003]

## RESULTS: HYGIENE IS NOT A RISK FACTOR FOR AD

Per-country ADadj values for GBD2016 plotted against WHO2004 values revealed a negative correlation (not presented), demonstrating that the datasets differ substantially. However, there was no evidence for systematic differences in the ways in which the WHO and GBD datasets perform age-adjustments and calculate DALYs (on which ADadj is based; not presented). We then plotted the key parameter used by Fox *et al.*, parasite stress, against the GBD2016 ADadj rates (Fig. 1). Whereas they observed a strong negative correlation, in which higher parasite burden was associated with reduced rates of AD (*r* = −0.61, *P* = <0.0001), we report a significant trend towards greater AD prevalence with increasing parasite burden (*r *=* *0.28, *P *=* *0.0003).

## CONCLUSION

The revised data provided by GBD2016 challenge the idea that hygiene is a risk factor for AD, and instead point to a positive association between parasite burden and AD. The major differences between the WHO2004 and GBD2016 datasets most likely reflect correction for underdiagnosis and under-reporting, one of the central goals of the GBD project [11]. This is an issue in less-resourced countries, and could explain a large part of the differences between the results of Fox *et al.* and those reported here. Of note, the ADadj metric (age-normalized DALYs per 100 000 population) used both by Fox *et al.* and here may be biased because technologically more advanced countries may have relatively extended lifespans as a result of better medical care, thus tending to selectively increase ADadj values in these countries. Nevertheless, compensation for this factor (i.e. by further downwards adjustment of AD rates in developed countries) would tend to increase, rather than decrease, the positive correlation between per-country AD rates and parasite stress.

In sum, this work challenges the global gradient of AD not only in relation to hygiene [1] but also in other more recent studies based on the WHO2004 dataset, such as those addressing dietary flavonoids [13] and vaccines [14]. We do not disagree with Fox *et al.* [1] that excessive hygiene might leave individuals at risk for inflammatory/infectious disorders such as AD, but the most recent data on global prevalences of AD no longer support this contention. Instead, a more direct mechanism may operate, and the positive relationship between parasite stress and AD is consistent with proposals that infection may be involved in the pathoetiology of the disease [2, 3], further borne out by the (modest) increase in dementia risk reported in patients with non-specific infectious disease e.g. Ref. [15]).

From an evolutionary perspective, the prevalence of AD may have been far lower for the majority of human history, perhaps reflecting shorter lifespans, and AD could thus be a ‘disease of civilization’ [16]. However, an unresolved question concerns why some developed countries such as Finland display high rates of AD, whereas others such as Singapore report much lower rates, a differential that cannot easily be ascribed to under-resourcing. Potential differential factors include genetic background [17], sunlight-dependent vitamin D synthesis [18], and diets rich in plant-derived antimicrobials (spices) [19] and/or nuts and essential oils (Mediterranean diet) [20]. Understanding the mechanisms underlying regional variations in disease rates including AD remains a priority topic.
